# Coating the surface of interconnected Cu_2_O nanowire arrays with HKUST-1 nanocrystals via electrochemical oxidation

**DOI:** 10.1038/s41598-023-39982-x

**Published:** 2023-08-24

**Authors:** Francesco Caddeo, Florian Himmelstein, Behzad Mahmoudi, Ana María Araújo-Cordero, Denis Eberhart, Haojie Zhang, Titus Lindenberg, Angelika Hähnel, Christian Hagendorf, A. Wouter Maijenburg

**Affiliations:** 1 Center for Innovation Competence SiLi-Nano, Martin Luther University Halle-Wittenberg, Karl-Freiherr-von-Fritsch-Straße 3, 06120 Halle (Saale), Germany; 2 Institute of Chemistry, Martin Luther University Halle-Wittenberg, Kurt-Mothes-Straße 2, 06120 Halle (Saale), Germany; 3 Institute of Physics, Martin Luther University Halle-Wittenberg, Heinrich-Damerow-Straße 4, 06120 Halle (Saale), Germany; 4 Fraunhofer Center for Silicon Photovoltaics CSP, Otto-Eißfeldt-Straße 12, 06120 Halle (Saale), Germany; 5 Present Address: Center for Hybrid Nanostructures (CHyN), Institute of Nanostructure and Solid State Physics, University of Hamburg, 22607 Hamburg, Germany; 6 Present Address: Max Planck Institute of Microstructure Physics, Weinberg 2, 06120 Halle (Saale), Germany

**Keywords:** Electrochemistry, Inorganic chemistry, Materials chemistry, Chemical synthesis

## Abstract

Controlling the crystallization of Metal–Organic Frameworks (MOFs) at the nanoscale is currently challenging, and this hinders their utilization for multiple applications including photo(electro)chemistry and sensors. In this work, we show a synthetic protocol that enables the preparation of highly homogeneous Cu_2_O@MOF nanowires standing on a conductive support with extensive control over the crystallization of the MOF nanoparticles at the surface of the Cu_2_O nanowires. Cu_2_O nanowires were first prepared via templated electrodeposition, and then partially converted into the well-known Cu-MOF HKUST-1 by pulsed electrochemical oxidation. We show that the use of PVP as a capping agent during the electrochemical oxidation of Cu_2_O into HKUST-1 provides control over the growth of the MOF nanocrystals on the surface of the Cu_2_O nanowires, and that the size of the MOF crystals obtained can be tuned by changing the concentration of PVP dissolved in the electrolyte. In addition, we propose the use of benzoic acid as an alternative to achieve control over the size of the obtained MOF nanocrystals when the use of a capping agent should be avoided.

## Introduction

Metal–Organic Frameworks (MOFs) are a class of crystalline nanoporous materials that hold high promises for a wide range of applications, including gas storage^1^, gas separation^2^, sensors^3^, drug delivery^4^ and heterogeneous (photo)catalysis^5–7^. In recent years, increasing efforts have been devoted in the MOF community towards the development of synthetic methods that enable tuning the MOF crystal growth at the nanoscale regime^8^, i.e. the so-called nano-MOFs, which include microwave^9^, sonochemical^10^, solvothermal^11^, microemulsion^12^ and droplet-based microfluidic synthesis^13^. The size control of MOF crystals at the nanoscale is particularly important in the field of heterogeneous catalysis, where the diffusion of reactive species through the MOF’s nanoporous architecture plays a very important role in terms of efficiency^14^.

Another class of synthetic methods that is considered highly promising in view of processability and large-scale preparation of MOFs is electrochemical synthesis^15–17^. Among all different electrochemical methods, including anodic oxidation^18^, reductive deprotonation^19^, galvanic replacement^20^ and electrophoretic deposition^21^, the anodic oxidation of a metallic substrate immersed in an electrolyte containing the appropriate organic linker is the most widely used, since it has several advantages including mild synthetic conditions, nontoxic solvents and short reaction times. Many well-known MOFs have been synthetized via anodic oxidation so far, including HKUST-1^22^, ZIF-8^23^, MIL-100^24^, MOF-5^25^ and UiO-66^26^.

In addition to metallic substrates, metal oxides could also be used as starting materials for the preparation of MOFs through anodic oxidation, provided that the metal cations can undergo further oxidation. For example, Cu^+^ ions in Cu_2_O can be further oxidized to Cu^2+^, favoring the formation of Cu-based MOFs. To the best of our knowledge, the electrochemical synthesis of Cu-based MOFs through the direct anodic oxidation of Cu_2_O has not been studied yet. However, it has been shown first by Schäfer et al.^27^ and then confirmed by our recent work^28^ that Cu_2_O is formed as an intermediate during the electrochemical conversion of metallic Cu into the MOF HKUST-1, and therefore we propose here that Cu_2_O could also be directly used as a starting material for the electrochemical preparation of Cu-based MOFs. Additionally, HKUST-1 was also prepared using techniques such as chemical-vapor deposition (CVD), solvothermal or sol–gel synthesis through the conversion of Cu-based precursors including Cu or CuO films and Cu(II) hydroxide^29–31^.

Cu_2_O nanostructures have been proposed as promising candidates for a number of applications, including photovoltaics^32^, photoelectrochemical water splitting^33^, lithium-ion batteries^34^, sensors^35^ and catalysis^36^. Cu_2_O nanowires in particular have received attention in the field of photoelectrochemical (PEC) water splitting^37^, since their shape allows efficient light absorption over their length, whereas the minority charge carriers can be collected across their diameter, effectively overcoming the problems related to the well-known mismatch between the light absorption depth and minority charge carrier diffusion length of this material^38,39^.

An easy and inexpensive way to obtain metal oxide nanowires is by templated electrodeposition within the pores of ion-track etched polycarbonate membranes. These commercially available membranes are fabricated by swift heavy ion irradiation and subsequent chemical etching, and can be obtained with tuneable pore diameters (from 20 nm to several micrometers), pore densities (1 to 10^10^ pores·cm^−2^) and different pore arrangements (from parallel arrays to highly interconnected pores)^40–42^. The highly interconnected arrangement in particular gives the possibility to obtain nanowires with a high aspect ratio (length/diameter up to 1000) due to the increased mechanical stability, with the length of the nanowires being tuned by the electrodeposition time^43,44^. Cu_2_O nanowires obtained via this technique were recently investigated as photocathodes for PEC water splitting^45^.

In our recent work, we introduced a synthetic protocol that combined the templated electrodeposition of Cu nanowires within the pores of ion-track etched membranes and their conversion into HKUST-1 nanowires via anodic oxidation^28^. In that work, Cu nanowires were electrochemically converted into MOFs while being enclosed within the polycarbonate membrane used as a template, therefore generating MOF nanowires with tuned diameters, since the crystallization of the MOF was constrained within the pores of the membrane.

In addition to the formation of nanowires that fully consist of HKUST-1, another interesting avenue is the preparation of MOF coatings on various nanostructures, therefore generating hybrid materials with synergistic properties. For example, Huang et al.^46^ developed a general strategy for the synthesis of ZIF-8 coatings via surface modification on various substrates, including carbon nanotubes (CNTs), SiO_2_, NiO and Fe_2_O_3_ nanostructures; Hu et al.^47^ showed the epitaxial growth of a 2D MOF on a pristine graphene surface for electrochemical applications; He et al.^48^ reported a ternary TiO_2_-Cu_2_O-HKUST-1 composite material synthesized through the in-situ growth of HKUST-1 on the TiO_2_-Cu_2_O surface, making use of part of the Cu_2_O as sacrificial copper source, which was tested for the photocatalytic reduction of CO_2_ to CH_4_; Zhan et al.^49^ prepared ZnO@ZIF-8 nanowires and tested their performance as a sensor for the detection of H_2_O_2_; Demirel et al.^50^ studied the electrochemical synthesis of HKUST-1 coatings on hollow copper fibers via anodic dissolution; Wu et al.^51^ prepared cubic Cu_2_O@HKUST-1 nanostructures via an in-situ conversion procedure and tested the material for the photocatalytic degradation of tetracycline hydrochloride. In addition to these examples, many more important papers and reviews have been published on this subject^17,52–56^.

In this work, we combined templated electrodeposition in polycarbonate membranes with subsequent anodic dissolution to prepare interconnected core–shell Cu_2_O@HKUST-1 nanowires standing on a conductive substrate. In addition, we studied the influence of PVP and benzoic acid as additives in the electrolyte used for the anodic dissolution to control the crystal growth of the obtained HKUST-1 nanoparticles at the surface of the Cu_2_O nanowires.

## Results and discussion

### Standing Cu_2_O nanowires via templated electrodeposition inside polycarbonate membranes

**Figure 1:**
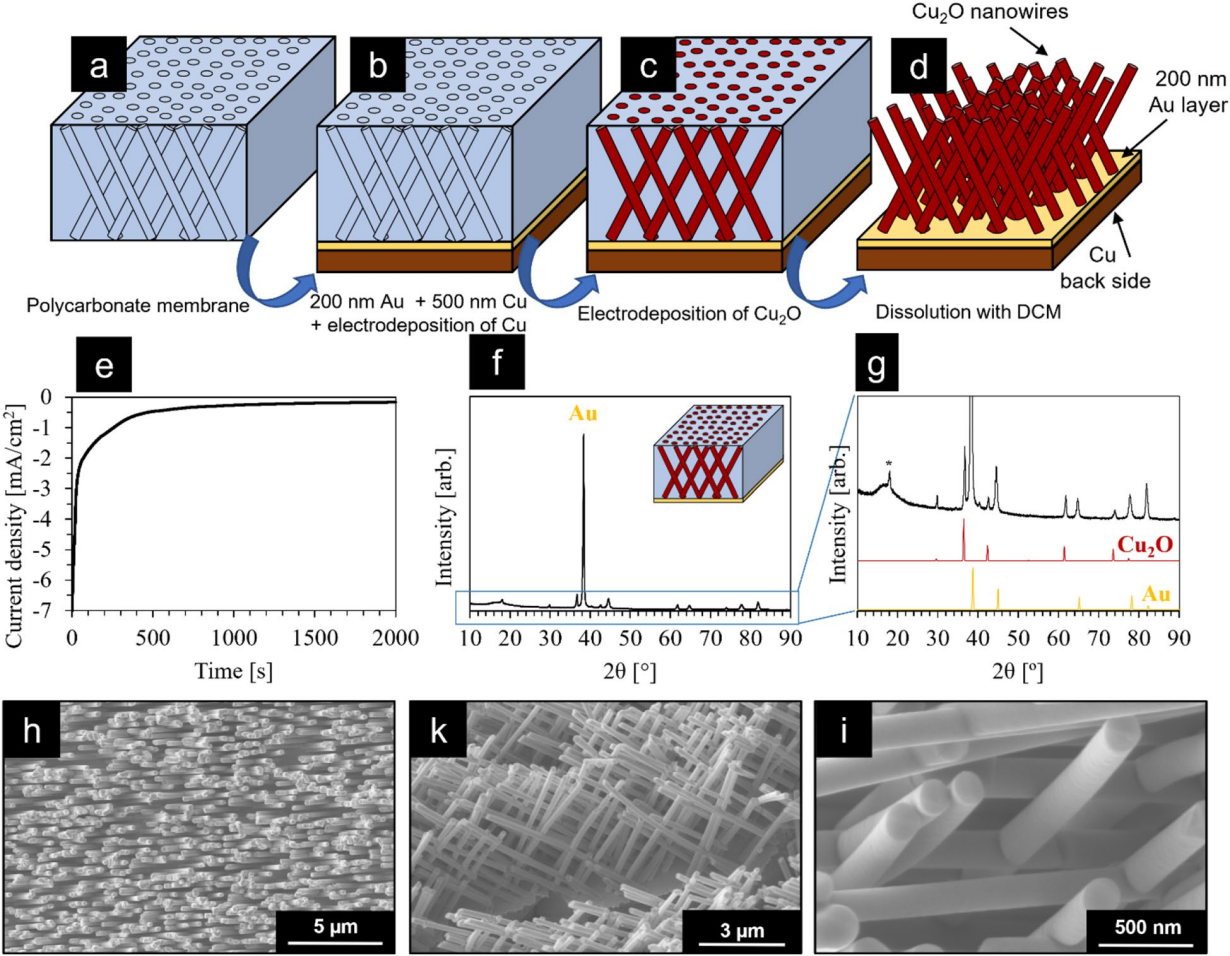
Preparation of interconnected Cu_2_O nanowire arrays. (**a**–**d**) Schematic representation of the synthetic steps used for the preparation of the Cu_2_O nanowires: (**a**) polycarbonate membrane; (**b**) sputtering of Au and Cu on one side of the membrane and electrodeposition of Cu on the same side; (**c**) electrodeposition of Cu_2_O inside the pores of the membrane; (**d**) dissolution of the membrane to obtain standing Cu_2_O nanowires; (**e**) chronoamperogram obtained during the electrodeposition of the Cu_2_O nanowires at − 0.4 V versus a Cu counter electrode; (**f**) XRD pattern of the Cu_2_O nanowires (Au reference card: ICSD code: 44,362; Cu_2_O reference card: ICSD code: 180,846) (with the inset showing a representation of the sample measured, i.e. Cu_2_O nanowires within the polycarbonate membrane, with only a 200 nm gold layer on the back side of the membrane); (**g**) enlarged area of the XRD pattern in (**f**); (**h**) top-view SEM image of the Cu_2_O nanowires; (**k**) side-view SEM image of the nanowires; (**i**) high-magnification SEM image of the same nanowires.

Cu_2_O nanowires standing on a conductive substrate were prepared by templated electrodeposition using commercially available polycarbonate membranes as the template. In Fig. 1a–d, a schematic representation of the process is shown, while photographs of the samples for each synthetic step are reported in Fig. S1, and SEM images of the bare polycarbonate membranes are reported in Fig. S2. As a first step, 200 nm of Au and 500 nm of Cu were consecutively deposited on one side of the polycarbonate membrane via sputtering (Fig. 1b and S1a,b). This sputtering step has a dual function: making one side of the membrane conductive and at the same time closing the pores of the membrane. Cu was chosen as a cheap and earth-abundant material for the back side of our electrodes, whereas Au was used as an intermediate layer for its good adhesion to the polycarbonate membrane. After sputtering, a thick layer (hundreds of micrometers) of metallic Cu was deposited on the conductive side of the membrane using a previously reported electrodeposition recipe^43^ (Fig. S1c) in order to obtain a mechanically stable electrode.

Once the back side of the electrode was ready, p-Cu_2_O was electrodeposited inside the pores of the membrane (Fig. 1c) following a previously reported procedure^45,57,58^, obtaining Cu_2_O nanowires. Here, the choice of the polycarbonate membrane determines the diameter of the nanowires, the nanowire density and their organization, and the electrodeposition time determines the nanowire length. For this study, we chose polycarbonate membranes with a pore diameter of 200 nm and a pore density of 5·10^8^ cm^−2^. Moreover, the membranes used have interconnected pores, which in turn generate interconnected nanowires as shown in Fig. 1c, k, i. For all of the samples presented in this study, we used an electrodeposition time of 2000s (~ 33 min), which generated nanowires with a length of ca. 10 µm, corresponding to an aspect ratio of 50. In this respect, it should be mentioned that previous works have been published on vertically grown one-dimensional arrays of nanowires, including the work by the Grätzel group on Cu_2_O nanowires^37^. However, such vertically aligned non-interconnected nanowires can be prepared only with a rather limited aspect ratio due to their intrinsic fragility. Therefore, in this work, we also highlight the importance of using templates with interconnected pores in order to obtain interconnected nanowire arrays with enhanced mechanical stability, allowing to obtain higher aspect ratios.

Once the electrodeposition was successfully carried out, the polycarbonate membranes were dissolved with dichloromethane (DCM), yielding standing Cu_2_O nanowires (Fig. 1d). A photograph of a sample after the dissolution step with DCM is given in Fig. S1d. Figure 1e shows a typical chronoamperogram that was recorded during the electrodeposition of the p-Cu_2_O nanowires within the pores of the membrane. At the first stage, the deposition proceeds at high current densities since the conductive Au back electrode allows a fast deposition. After ~ 300 s, the current density decreases considerably due to the deposition happening on top of the already deposited semiconducting Cu_2_O nanowires, reaching a steady-state current density of ca. 200 µA cm^-2^.

The XRD pattern in Fig. 1f,g refers to the electrodeposited Cu_2_O nanowires inside a membrane with only sputtered Au as the back contact and without the additional Cu deposited on the back side, as shown in the inset in Fig. 1f. The XRD pattern therefore shows Cu_2_O reflections that can only originate from the electrodeposition inside the pores. Apart from a large contribution at ⁓ 16° coming from the amorphous polycarbonate (Fig. S3) and the peak indicated with an asterisk that originates from the Teflon tape used to fix the sample onto the XRD sample holder (Fig. S4), all other XRD reflections could be assigned to either Au or Cu_2_O^59,60^.

Figure 1h,i show SEM images of the as-prepared Cu_2_O nanowires after dissolution of the polycarbonate membrane in DCM. The low-magnification SEM image in Fig. 1h shows that the samples are highly homogeneous with the nanowires growing within all pores of the membrane. Figure 1k shows a side-view SEM image of the nanowires and Fig. 1i is a high-magnification SEM image that clearly shows the very smooth surface of the Cu_2_O nanowires, indicating the high crystallinity of the deposited nanowires.

### Partial conversion of Cu_2_O into HKUST-1

In the next step, the Cu_2_O nanowires were partially converted into the well-known Cu-based MOF HKUST-1 (Cu_3_(BTC)_2_; BTC = 1,3,5-benzenetricarboxylic acid), with the aim to obtain Cu_2_O nanowires decorated with MOF nanoparticles. This was achieved via electrochemical oxidation of the Cu_2_O nanowires and simultaneous formation of HKUST-1, with a special emphasis on the fine-tuning of a number of synthetic parameters, including the electrochemical oxidation process, the presence of appropriate additives in the electrolyte and the reaction time. For the electrochemical oxidation, a 2-electrode setup was used with the Cu_2_O nanowires presented in the previous section as the working electrode and a Cu wire as the counter electrode.

First of all, we decided to employ a pulsed electrochemical oxidation process, in which the potential was alternated 100 times between 2.5 and 0.0 V (vs. a Cu counter electrode), which were applied for 0.1 and 0.5 s, respectively. This choice is motivated by our goal to obtain a composite material where Cu_2_O nanowires are decorated with MOF nanoparticles in a core–shell type structure. We imagine that while Cu^+^ ions from Cu_2_O are oxidized to Cu^2+^ during the pulses at 2.5 V, a certain time is needed for these ions to react with the BTC molecules and to form MOF nanocrystals at the surface of the Cu_2_O nanowires. Additionally, in one of our previous studies, we performed the complete electrochemical conversion of Cu nanowires into HKUST-1 using the same electrolyte, but by applying a continuous potential 2.5 V versus Cu for 2 h, and we verified that the obtained HKUST-1 nanowires were stable in these oxidizing conditions^28^.

**Figure 2:**
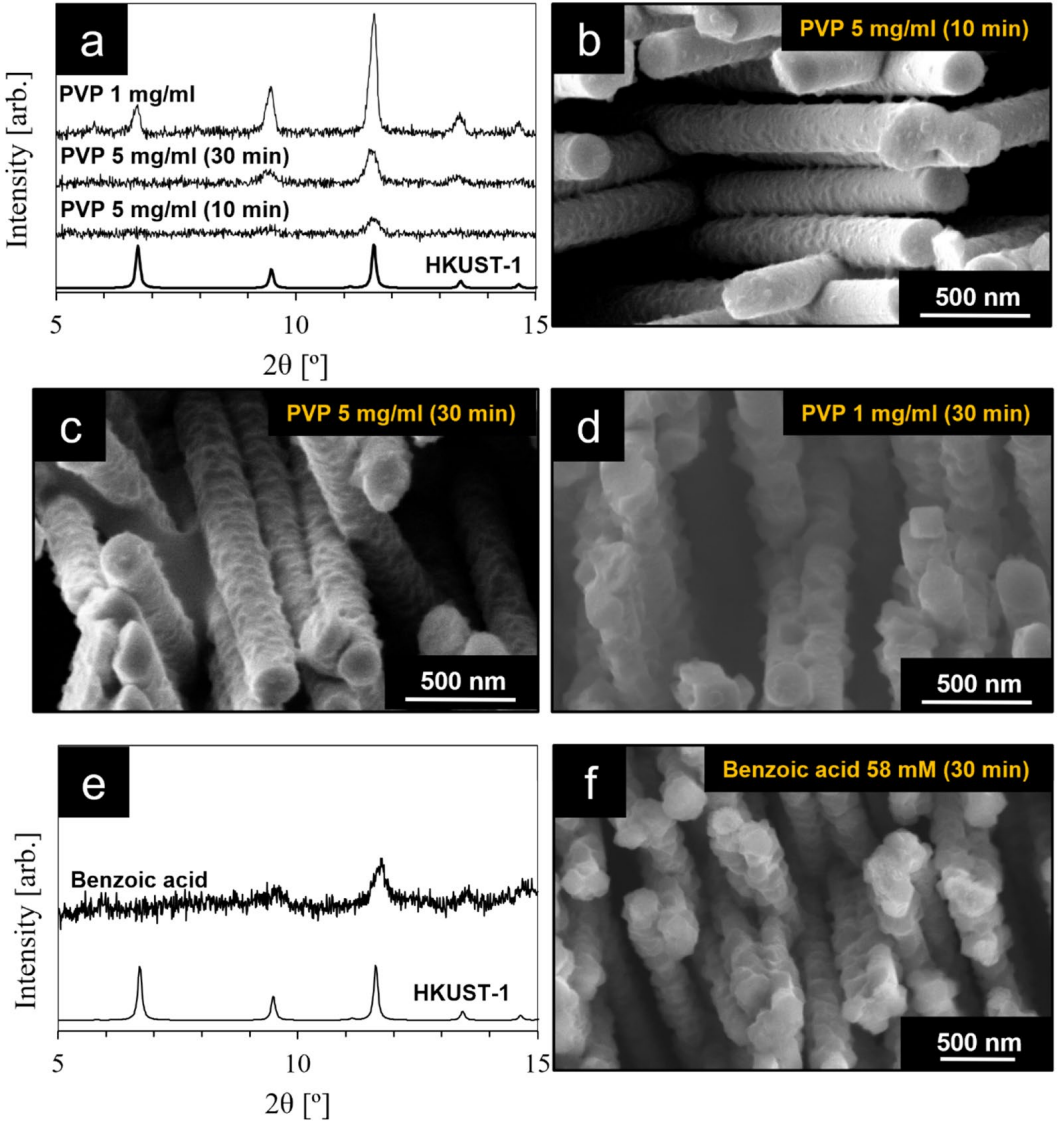
Effect of additives and reaction time on the partial conversion of Cu_2_O into HKUST-1. (**a**) XRD patterns of the nanowires after partial conversion of Cu_2_O into HKUST-1 in the presence of PVP as a capping agent; (**b**) SEM image of the Cu_2_O@HKUST-1 nanowires obtained with a PVP concentration of 5 mg mL^−1^ and an overall reaction time of 10 min; (**c**) SEM image of Cu_2_O@HKUST-1 nanowires obtained with a PVP concentration of 5 mg mL^−1^ and an overall reaction time of 30 min; (**d**) SEM image of Cu_2_O@HKUST-1 nanowires obtained with a PVP concentration of 1 mg mL^−1^ and an overall reaction time of 30 min; (**e**) XRD pattern and (**f**) SEM image of Cu_2_O@HKUST-1 nanowires obtained using a benzoic acid concentration of 58 mM and an overall reaction time of 30 min.

Moreover, it should be highlighted that the use of appropriate additives dissolved in the electrolyte, i.e. PVP and benzoic acid, also greatly contributed to the successful formation of MOF nanoparticles at the surface of the Cu_2_O nanowires. In fact, when performing the electrochemical conversion of Cu_2_O using an ethanolic solution of BTC without any additive, very large HKUST-1 crystals were found that do not adhere to the surface of the Cu_2_O nanowires, as shown in the SEM images in Fig. S5. A similar result was obtained in a recent study by Luo et al., during the electrochemical conversion of Cu nanowires into HKUST-1 in the absence of appropriate additives^61^. Therefore, in order to control the growth of the MOF crystals during the electrochemical oxidation, we first explored the influence of PVP dissolved in the electrolyte using two different concentrations, i.e. 5 and 1 mg mL^−1^, and as can be seen in Fig. 2b–d, this resulted in the controlled formation of MOF nanocrystals around the Cu_2_O nanowires. Indeed, PVP is a well-known capping agent, which is broadly used in nano-chemistry to control the growth of different kinds of nanoparticles^62,63^. Moreover, while PVP was rarely used in previous studies during the synthesis of HKUST-1, it must be highlighted that Li et al.^64^ showed the PVP-mediated synthesis of HKUST-1 octahedron nanoparticles with sizes ranging from 50 to 500 nm.

Another important parameter of the conversion process is the overall reaction time. In fact, it is known from previous reports that Cu_2_O also converts into Cu_3_(BTC)_2_ without applying any electrochemical potential, in which dissolved oxygen from the air acts as the oxidizing agent^54^. Therefore, the time that the Cu_2_O nanowires were immersed in the electrolyte represents an important parameter, as the conversion reaction also proceeds before and after the pulsed electrochemical process. Here, we show results corresponding to overall reaction times of 10 and 30 min, of which only 1 min was used for the actual electrochemical oxidation, with the oxidative potential of 2.5 V only being applied for 10 s.

Fig. S6 shows a typical chronoamperogram of the process, with an enlarged area of the chronoamperogram being reported in the inset of the figure. A current density of ca. 70 µA cm^−2^ was generated during the pulses at the oxidative potential of 2.5 V, corresponding to the oxidation of Cu^+^ into Cu^2+^. Figure 2a shows XRD patterns of the materials obtained using PVP as a capping agent together with the XRD pattern of HKUST-1 calculated from single crystal data^65^. Due to the small size of the obtained HKUST-1 nanoparticles and their presence in a small quantity in terms of overall mass, the reflections from the MOF crystals are very weak compared to the ones associated to Cu_2_O, Cu and Au. Therefore, Fig. 2a shows the XRD patterns in the 2θ range of 5–15°, where the most prominent reflections corresponding to HKUST-1 are present, whereas the entire range is shown in Fig. S7. All XRD patterns show the presence of HKUST-1. In the case of the sample obtained using PVP with a concentration of 5 mg mL^−1^ and a total reaction time of 10 min, only the most intense reflection at 2θ = 11.7° (full width at half maximum (FWHM) = 0.36°, corresponding to a crystallite size of ca. 27 nm calculated with the Debye–Scherrer method) is visible due to the small size and small amount of the MOF nanoparticles (Fig. 2b). With increasing the overall reaction time to 30 min, this reflection becomes more intense, and additionally we can observe the appearance of the second most intense reflection centered at 2θ = 9.6°. These reflections are broad with a FWHM of 0.27° for 2θ = 11.7° and 0.34° for 2θ = 9.6°, corresponding to a crystallite size of ca. 38 nm and 29 nm, respectively, suggesting crystal growth when compared to the sample obtained after 10 min, as also observed in Fig. 2c. The XRD pattern of the material obtained by decreasing the concentration of PVP to 1 mg·mL^-1^ and by keeping an overall reaction time of 30 min shows all the reflections corresponding to HKUST-1 in this 2θ range. Moreover, the peaks appear sharper, with the FWHM of the peak at 2θ = 11.7° being 0.18°, which corresponds to a crystallite size of ca. 64 nm calculated with the Debye–Scherrer method. This enhancement of size of the HKUST-1 nanoparticles is in agreement with the obtained SEM image from this sample (Fig. 2d).

When taking a closer look at the SEM images of the Cu_2_O@HKUST-1 nanowires obtained using PVP with a concentration of 5 mg mL^−1^ after a total reaction time of 10 min (Fig. 2b) and 30 min (Fig. 2c), it can be seen that the Cu_2_O nanowires appear discontinuously decorated with the MOF nanoparticles at their surface along the full nanowire length. Increasing the reaction time from 10 to 30 min resulted in larger nanoparticles that appear to cover most of the surface of the Cu_2_O nanowires, as shown in Fig. 2c. These evidences agree with the XRD patterns (Fig. 2a) and with the presence of MOF nanoparticles as a result of the electrochemical oxidation of Cu_2_O.

Figure 2d shows the nanowires obtained after the electrochemical oxidation in the presence of PVP with a lower concentration, i.e. 1 mg mL^−1^. As expected^62^, and in agreement with the XRD pattern, the MOF nanoparticles at the surface of these Cu_2_O nanowires appear larger in size compared to the ones obtained using a higher amount of PVP in the electrolyte. While the size of the nanoparticles obtained using 5 mg mL^−1^ of PVP is in the range of 10–50 nm, the ones obtained using 1 mg mL^−1^ of PVP are overall larger, reaching sizes of more than 100 nm in some cases. This trend is also in agreement with the XRD patterns, since the MOF nanoparticles obtained with a lower concentration of PVP generated sharper reflections, which is also highlighted by the calculated FWHM values. We therefore conclude that the size of the MOF crystals can be tuned by changing the concentration of PVP dissolved in the electrolyte solution and the overall reaction time.

However, one of the drawbacks of using capping agents with a long alkyl chain, such as PVP, is that the capping agent molecules might stay bonded to the surface^63^. This is a major drawback for some applications, such as catalysis or sensors, where the chemical properties of the nanoparticle’s surface play a major role. We therefore explored the possibility of substituting PVP with benzoic acid, which is a well-known modulator in the MOF field^66^. Modulators are typically molecules that are similar to the organic linkers used for the specific MOF synthesis, but bearing only one functional group (i.e. carboxylate in our case). The modulator therefore reacts with the metal oxo-cluster during MOF synthesis but does not lead to the formation of a MOF, since it lacks the additional carboxylate functional group. Therefore, modulators are useful to slow down the dissolution–recrystallization mechanism, leading in some cases to the formation of single crystalline MOF particles^67^. However, they can also be used to slow down the crystal growth rate^68^. With this intention, we explored the use of benzoic acid instead of PVP, in order to obtain a similar control over the crystal size of the MOF nanoparticles during the electrochemical oxidation, while at the same time keeping the surface of the MOF crystals free of any capping agent.

Figure 2f shows the nanowires that were obtained by performing the conversion reaction in the presence of 58 mM benzoic acid (BA), which corresponds to a BTC:BA molar ratio of 1:10. Also in this case, the nanowires appear covered with MOF nanoparticles, which is also confirmed by the XRD pattern shown in Fig. 2e. Compared with the samples obtained using PVP, the nanowires converted using benzoic acid appear more uniformly covered with MOF nanoparticles. However, in this sample we also noticed the presence of large MOF crystals separated from the nanowires, as shown in Fig. S8, together with the MOF nanoparticles surrounding the Cu_2_O nanowires. These larger MOF particles are similar to the case in Fig. S5 where no additives were used. The influence of the concentration of benzoic acid on the morphology of the resulting MOF nanoparticles was also studied and the results are reported in Fig. S9.

In order to rule out the possibility that the nanoparticles shown in Fig. 2b–d,f could originate from a different material than HKUST-1, we performed a series of “control syntheses”, in which the same electrochemical conversion process was performed using an electrolyte with the presence of either PVP or benzoic acid, but with the absence of BTC, which is needed to form the MOF particles. The SEM characterization of these samples is reported in Figure S10 and shows that indeed no nanoparticles are formed at the surface of the Cu_2_O nanowires when the synthesis is performed in the absence of BTC in the electrolyte, which further confirms the successful synthesis of Cu_2_O@HKUST-1 nanowires.

**Figure 3:**
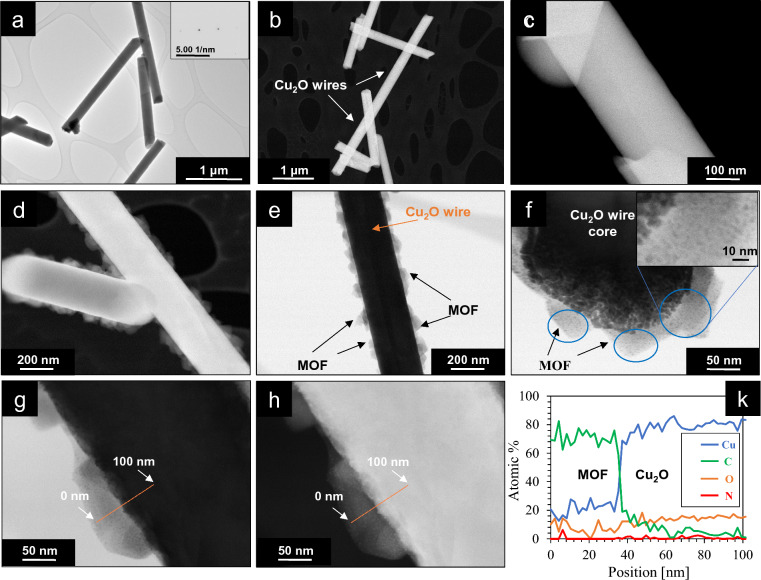
TEM characterization of the Cu_2_O@HKUST-1 nanowires. (**a**) BF-TEM and (**b**) LAADF-STEM images of Cu_2_O nanowires before conversion; inset in (**a**): SAED showing crystallinity; (**c**) higher magnification image showing the smooth surface of a Cu_2_O nanowire; (**d**) LAADF-STEM image of Cu_2_O@HKUST-1 nanowires obtained using PVP with a concentration of 5 mg mL^−1^ and a reaction time of 30 min; (**e**) and (**f**) BF-TEM images of a Cu_2_O@HKUST-1 nanowire obtained using the same conditions, with the inset in (**f**) showing an enlarged area; (**g**) BF-TEM and (**h**) LAADF-STEM images of a similar Cu_2_O@HKUST-1 nanowire at high magnification and (**k**) corresponding EDX line-scan across the MOF-Cu_2_O interface showing an estimation of the amount of C, Cu, N and O (in atomic %) by Cliff-Lorimer quantification.

The Cu_2_O and Cu_2_O@MOF nanowires were also characterized by transmission electron microscopy (TEM), low-angle annular dark-field scanning transmission electron microscopy (LAADF-STEM) and energy-dispersive X-ray spectroscopy (EDX) (Fig. 3). Figure 3a shows a bright-field (BF) TEM image of the Cu_2_O nanowires before their conversion into a MOF and Fig. 3b,c show LAADF-STEM images of Cu_2_O nanowires from the same sample. The images highlight the very smooth surface of the nanowires, both at the micrometer and at the nanometer scale. The crystallinity of the nanowires, which was already demonstrated by the XRD pattern presented in Fig. 1g, is confirmed by selected area electron diffraction (SAED) as shown in the inset in Fig. 3a.

Figure 3d shows a LAADF-STEM image of the Cu_2_O@MOF nanowires obtained using PVP with a concentration of 5 mg mL^−1^ and a reaction time of 30 min, while Fig. 3e,f show BF-TEM images of the same sample. In agreement with Fig. 2c, the nanowires appear discontinuously covered by MOF nanoparticles with sizes in the range of 10–50 nm. At high magnification, the interface between the MOF particles and the Cu_2_O nanowire appears textured with a fine-grinded zone with grain sizes in the range of 10–20 nm (Fig. 3f). These crystalline nanoparticles appear embedded in a light matrix, and most likely result from the incomplete oxidation process of the Cu_2_O, with the MOF particles growing around these nanoparticles as soon as the Cu^2+^ ions were available. The MOF particles are clearly visible due to their strong contrast with the Cu_2_O nanowires. A close inspection reveals that very small particles (< 5 nm, visible as small dark dots in the regions indicated by the blue circles and enlarged in the inset in Fig. 3f) are embedded within the MOF nanocrystals, which might be small Cu_2_O nanoparticles that were not completely converted into MOF. Figure 3g,h show BF-TEM and LAADF-STEM images, and the corresponding EDX line scan across the MOF-Cu_2_O interface (orange line in Fig. 3g,h) with the atomic quantification of the different elements present is reported in Fig. 3k. The EDX analysis confirms that the particles surrounding the Cu_2_O wires correspond to the MOF, since the amount of C detected quickly drops at the interface, while the signal for Cu increases at the same point. The signal for C drops almost to zero, since a very small amount of C atoms are present in the Cu_2_O and the sample substrate, whereas the amount of Cu is approximately 20 at% inside the MOF crystal, corresponding with the presence of Cu in the Cu_3_(BTC)_2_ nanoparticle. Similar results were obtained in the case of the Cu_2_O@HKUST-1 nanowires synthesized using benzoic acid instead of PVP, of which the LAADF-STEM and EDX characterization is reported in Fig. S11.

Additional X-ray photoelectron spectroscopy (XPS) characterization is reported in Fig. S12. The Cu 2p spectra show the presence of Cu^+^ and Cu^2+^ oxidation states (peaks centered at 932 eV and 934 eV, respectively) for all samples, independently on the presence of MOF particles around the Cu_2_O nanowires. The presence of Cu^2+^ for the case of pristine Cu_2_O nanowires can be attributed to the oxidation of their surface in air^69^.

In summary, we have shown that the electrochemical synthesis of MOFs can be extended to the preparation of hybrid materials where MOF nanoparticles are grown at the surface of metal oxide nanostructures, such as Cu_2_O@HKUST-1 nanowire arrays standing on a support. Our synthesis method addresses two major challenges: (i) controlling the growth of MOF nanoparticles, which is achieved through electrochemical synthesis combined with the use of the appropriate additives, and (ii) the integration of MOFs in a nanoscale electrochemical device, which is achieved by the formation of the MOF during electrochemical oxidation of the parent nanostructure. We believe that, through this simple electrochemical method, many more MOFs could be integrated on various nanostructures, and that these hybrid materials could find application in fields such as gas-phase photocatalysis and for photoactive gas sensors, where the photoactivity of the metal oxides could be coupled with the gas absorption properties typical of MOFs. Recent studies in this direction show that HKUST-1 could indeed be used in sensors for the detection of CO_2_ or the relative humidity^70,71^.

## Conclusions

Here, we presented the preparation of Cu_2_O@HKUST-1 nanowires standing on a conductive support, which opens up the possibility to use such hybrid nanostructures in sensing and gas-phase photocatalysis. These composite nanowires were prepared by coupling two established synthesis strategies: (1) templated electrodeposition of Cu_2_O nanowires in ion-track membranes with (2) subsequent pulsed electrochemical oxidation of the Cu_2_O to obtain HKUST-1 nanocrystals at the surface of the Cu_2_O nanowires. Furthermore, we showed that the crystallization of the MOF can be controlled at the nanoscale using PVP as a capping agent, and that the size of the MOF nanocrystals can be tuned by changing the concentration of PVP in the electrolyte. We also showed that the crystallization of the MOF nanocrystals at the surface of the Cu_2_O nanowires can also be achieved by using benzoic acid as a modulator instead of PVP. The method allows the integration of MOF nanoparticles on a metal oxide based electrochemical device, and can be potentially extended to other MOFs.

### Experimental details

All chemicals used were purchased from commercial sources and used without further purification, namely: sodium hydroxide (NaOH, Carl Roth, ≥ 98%), ethanol (EtOH, CH_3_CH_2_OH, Chemsolute, 99.9%), dichloromethane (DCM, CH_2_Cl_2_, Chemsolute, 99.9%), copper sulfate pentahydrate (CuSO_4_·5H_2_O, Chemsolute, ≥ 99.0%), 1,3,5-benzenetricarboxylic acid (BTC, Alfa Aesar, 98%), sulfuric acid (H_2_SO_4_, Carl Roth, 95–98%), DL-lactic acid (C_3_H_6_O_3_, Fisher Scientific, 90%), polyvinylpyrrolidone K90 (PVP, (C_6_H_9_NO)_n_, Carl Roth, pure), benzoic acid (C_7_H_6_O_2_, Carl Roth, ≥ 99.5%), polycarbonate membranes (it4ip, interconnected pores, 0.2 μm diameter, pore density: 5·10^8^ cm^−2^, 25 μm thickness).

#### Preparation of the Cu_2_O nanowires

Polycarbonate membranes were first coated on one side with a 200 nm thick layer of Au and a 500 nm thick layer of Cu using a Leica EM ACE 600 sputter coater. After sputtering, metallic Cu was electrodeposited on the sputtered side of the polycarbonate membrane by adapting a previously published procedure^26^. In short, an aqueous solution containing 1.0 M CuSO_4_ and 0.2 M H_2_SO_4_ was heated to 60 °C, and the Cu was deposited by applying a potential of − 0.1 V for 7200 s (2 h) in a two-electrode setup versus a Cu wire that was used as the counter electrode. In the next step, p-Cu_2_O nanowires were electrodeposited by exposing the non-sputtered side of the membrane to the electrolyte, therefore allowing the electrolyte to enter the pores of the membrane. For the electrodeposition, a previously published procedure was used^45,58,72^. Briefly, an aqueous solution containing 0.4 M CuSO_4_ and 3 M lactic acid at pH = 11 was used as the electrolyte, and − 0.4 V was applied versus a Cu wire used as the counter electrode in a 2-electrode setup, for a total electrodeposition time of 2000s (~ 33 min). The pH of the solution was measured with a calibrated pH meter (Accumet AB150pH, Fisher Scientific). Here, the use of a high concentration of lactic acid in the solution is needed to avoid the precipitation of Cu(OH)_2_ at a pH of 11. As also reported in ref. 58, the Cu^2+^ ions are stabilized via the formation of copper lactate with the formula Cu(CH_3_CHOHCOO)_2_. After each electrodeposition, the samples were washed with distilled water, and finally, the polycarbonate membrane was dissolved in DCM.

#### Partial conversion of Cu_2_O into HKUST-1

The obtained Cu_2_O nanowires were partially converted into HKUST-1 in the form of nanoparticles that were grown at the surface of the Cu_2_O nanowires via a pulsed electrochemical oxidation method. As the electrolyte, a solution containing 5.8 mM BTC in 50/50 (v/v) EtOH/water was used, wherein either PVP or benzoic acid was also dissolved as either a capping agent or a modulator, respectively. In the case of PVP, the concentrations used were 5 and 1 mg mL^−1^. In the case of benzoic acid, we used 2.9, 5.8, 11.6, 58 and 116 mM and the best results were obtained with a concentration of 58 mM, corresponding to a molar ratio of BTC:benzoic acid of 1:10. Again, a two-electrode setup with a Cu wire as the counter electrode was used, and the electrochemical oxidation was performed using pulsed electrochemistry, during which a pulse of + 2.5 V versus Cu for 0.1 s was alternated with a second pulse of 0.0 V versus Cu for 0.5 s, and this was repeated for a total of 100 times, corresponding to a net oxidation time of 10 s at 2.5 V. However, the electrode was immersed inside the electrolyte for a total of either 10 or 30 min, meaning that a resting time of 5 or 15 min, respectively, was applied before and after the electrochemical conversion. All electrodepositions and electrochemical oxidation processes were carried out using an Autolab PGSTAT204 potentiostat (Metrohm).

#### Characterization

X-ray diffraction (XRD) measurements were performed using a Bruker D2 powder diffractometer equipped with a Cu Kα source. SEM images were obtained using a FEI Versa 3D DualBeam. The samples for TEM, LAADF-STEM and EDX characterization were prepared by scraping off the Cu_2_O and Cu_2_O@MOF nanowires on a Au-mesh covered with an ultrathin C-support film. The characterization was performed using a FEI TEM/STEM TecnaiG2 F20 Super Twin (acceleration voltage: 200 kV; HRTEM resolution: 0.24 nm; STEM-HAADF resolution: 0.19 nm; Thermo-Fisher Scientific/FEI Inc. equipped with an EDAX Si(Li)-detector (EDAX Inc.)). XPS measurements were carried out using a Thermo Scientific K-alpha X-ray photoelectron spectrometer system equipped with a monochromatic Al Kα X-ray source. All spectra were calibrated with a reference of C1s (248.8 eV).

### Supplementary Information

Supplementary Figures.
