# Supplementary material for: Coating the surface of interconnected Cu2O nanowire arrays with HKUST-1 nanocrystals via electrochemical oxidation
Source: Sci Rep. 2023 Aug 24;13:13858. doi: 10.1038/s41598-023-39982-x (PMC10449819; doi:10.1038/s41598-023-39982-x)
Supplement: Supplementary file 1 — Supplementary Figures. [file 41598_2023_39982_MOESM1_ESM.pdf]

Coating the surface of Interconnected Cu_2O Nanowire Arrays with HKUST-1 Nanocrystals via Electrochemical Oxidation

Francesco Caddeo[1,2], Florian Himmelstein[1,2], Behzad Mahmoudi[1,2], Ana Maria Araujo-Cordero[1,2], Denis Eberhart[1,2], Haojie Zhang[1,3], Titus Lindenberg[1,2], Angelika Hähnel[4], Christian Hagendorf[4] and A. Wouter Maijenburg[1,2*]

[1] Center for Innovation Competence SiLi-nano, Martin Luther University Halle-Wittenberg, Karl-Freiherr-von-Fritsch-Straße 3, 06120 Halle (Saale), Germany

[2] Institute of Chemistry, Martin Luther University Halle-Wittenberg, Kurt-Mothes-Straße 2, 06120 Halle (Saale), Germany.

[3] Institute of Physics, Martin Luther University Halle-Wittenberg, Heinrich-Damerow-Straße 4, 06120 Halle (Saale), Germany.

[4] Fraunhofer Center for Silicon Photovoltaics CSP Otto-Eißfeldt-Straße 12, 06120 Halle (Saale), Germany

- Supporting Information -

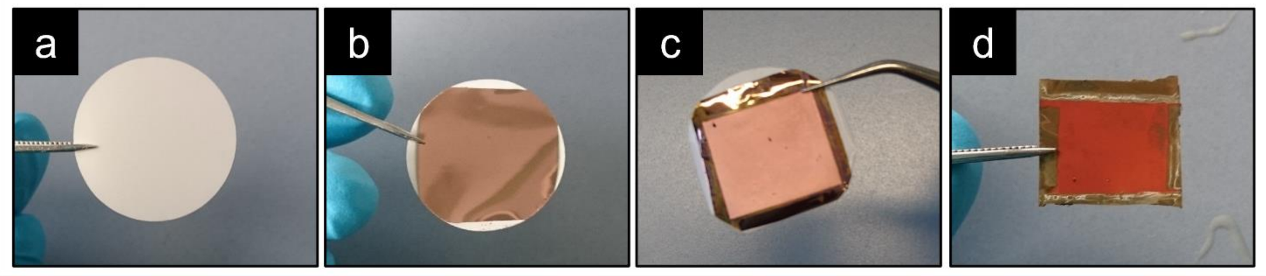

Figure S1. Photographs of the samples during the preparation of the Cu_2O nanowires: (a) polycarbonate membrane; (b) polycarbonate membrane after sputtering of 200 nm of Au and 500 nm of Cu on the back side; (c) polycarbonate membrane after electrodeposition of Cu on the back side; (d) front side after electrodeposition of Cu_2O electrodeposition and removal of the template with dichloromethane (DCM).

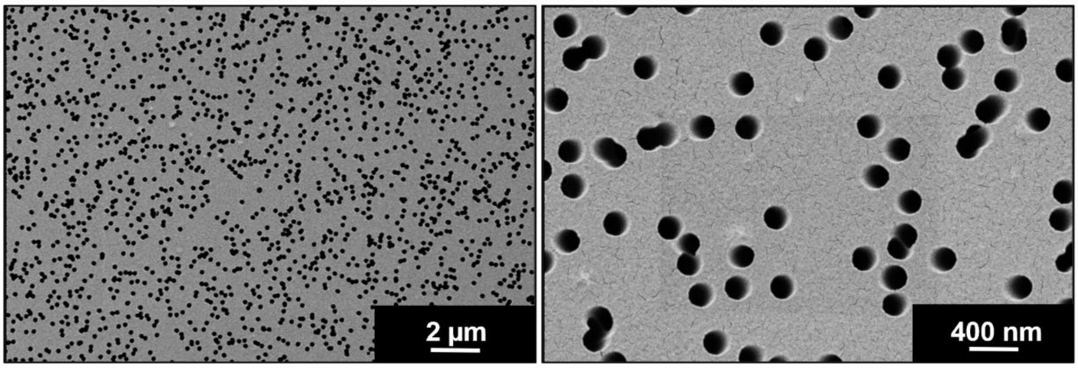

Figure S2. SEM images of the bare polycarbonate membranes.

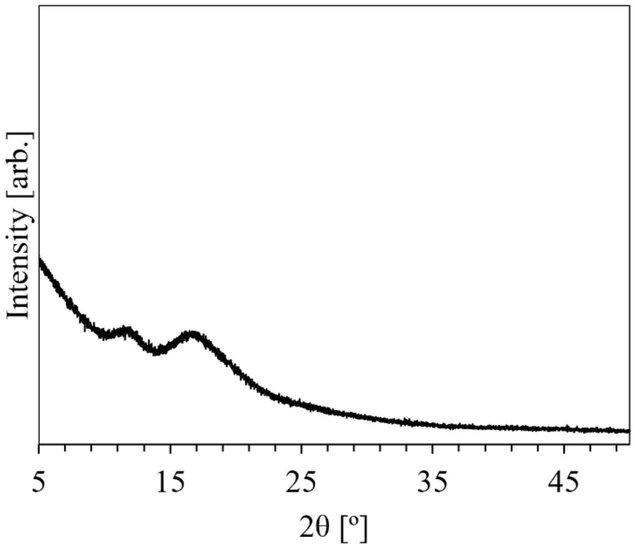

Figure S3. XRD pattern of the polycarbonate membrane.

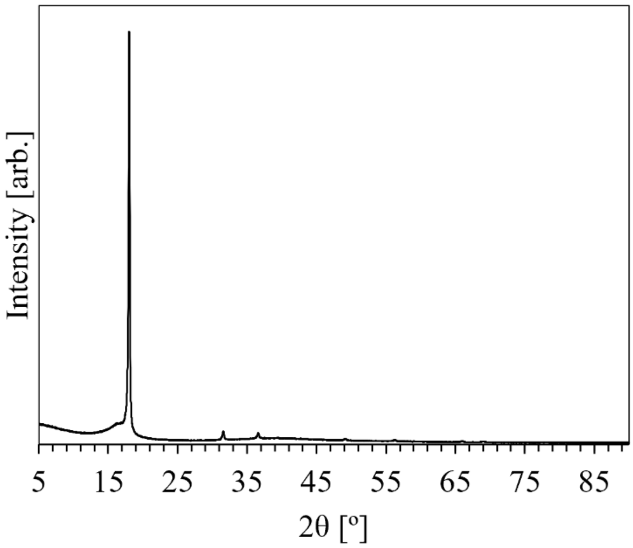

Figure S4. XRD pattern of the Teflon tape used to fix the sample onto the XRD sample holder.

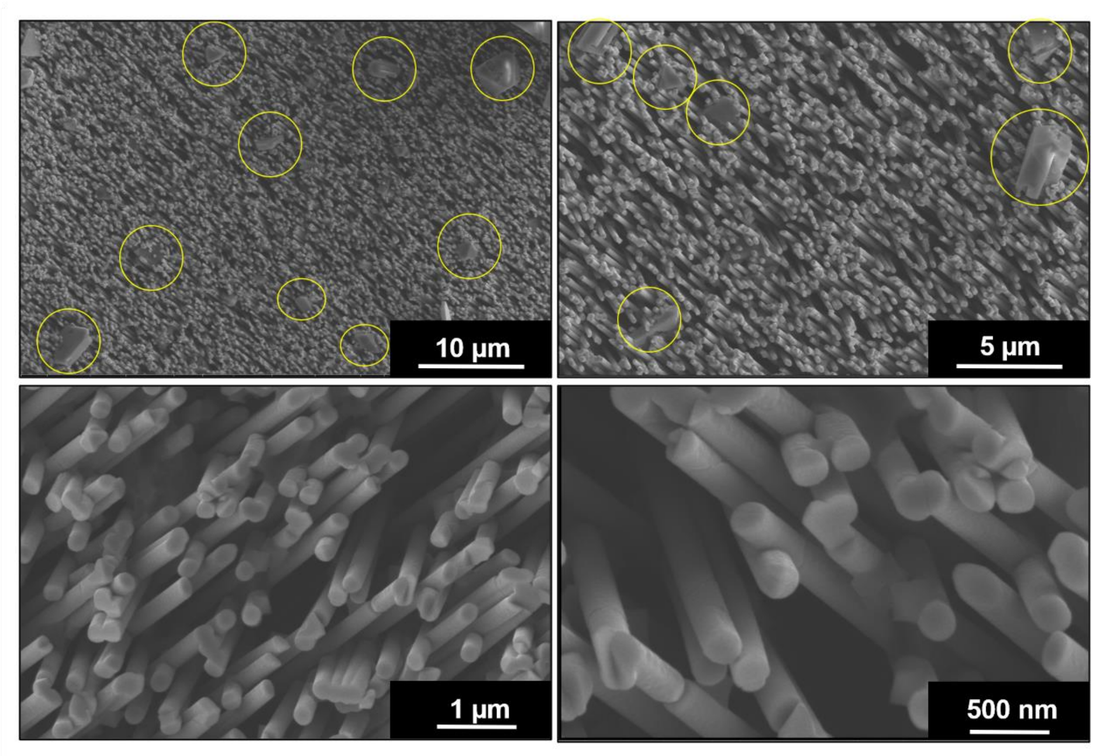

Figure S5. SEM images of a sample obtained after pulsed electrochemical conversion of Cu_2O nanowires into HKUST-1 without the use of additional additives such as PVP or benzoic acid. As highlighted by the yellow circles, in this case the MOF HKUST-1 forms as very large crystals.

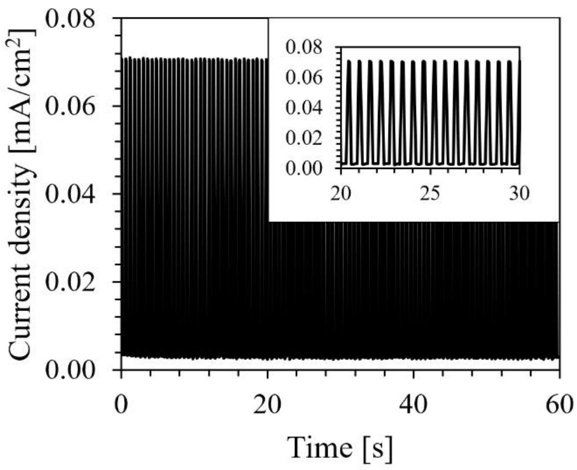

Figure S6. Chronoamperogram of a typical pulsed electrochemical oxidation.

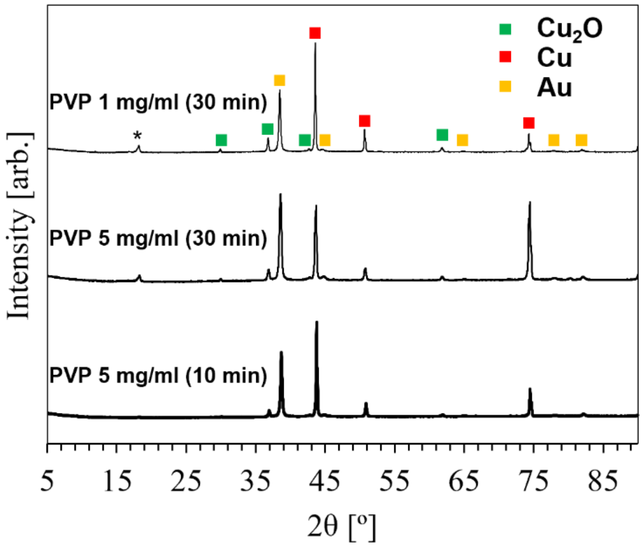

Figure S7. Full XRD patterns of the electrodes with Cu_2O@HKUST-1 nanowires, measured over a wide 2θ range from 5° to 90°; * corresponds to a reflection generated by the Teflon tape used to fix the sample on top of the XRD sample holder (**Figure S4**).

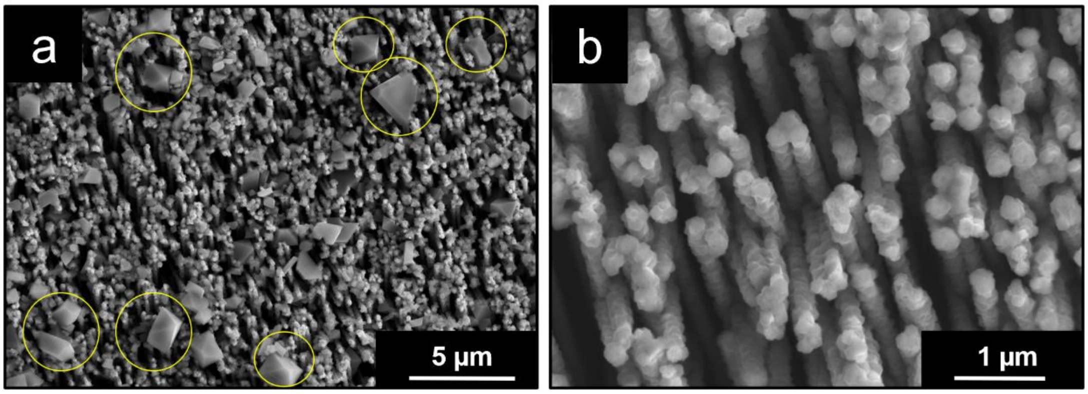

Figure S8. SEM images of the Cu_2O@HKUST-1 nanowires obtained using benzoic acid during the electrochemical conversion, showing (a) the formation of larger MOF crystals (yellow circles) together with (b) the MOF nanocrystals coating the nanowires.

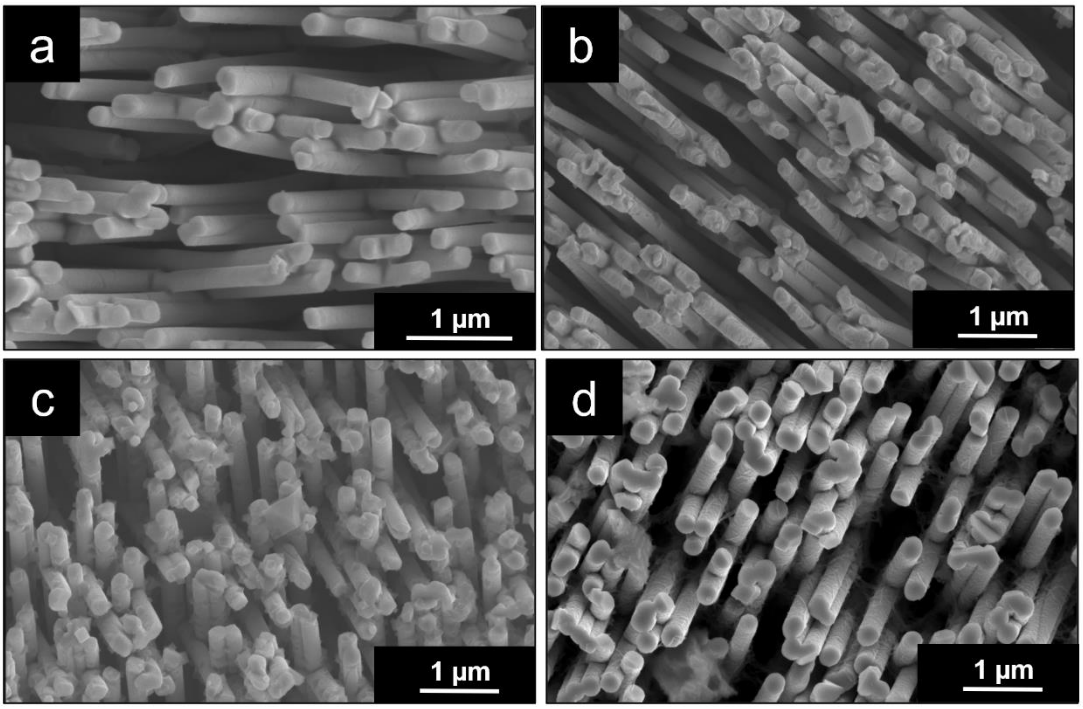

Figure S9. SEM images of the Cu_2O@HKUST-1 nanowires obtained using benzoic with concentrations of (a) 2.9 mM; (b) 5.8 mM; (c) 11.6 mM; (d) 116 mM. An SEM image of a sample obtained with an optimized concentration of 58 mM is reported in the main manuscript, **Figure 3a**. At low benzoic acid concentrations, MOF nanoparticles are not formed at the surface of the Cu_2O nanowires, but rather form big, detached particles, similarly to what is shown in **Figure S5** for the case of no additives. At high concentrations, such as for the case of 116 mM reported in (d), the MOF nanoparticles do not form, but a reticulated nanostructure is observed around the nanowires, which was not investigated further.

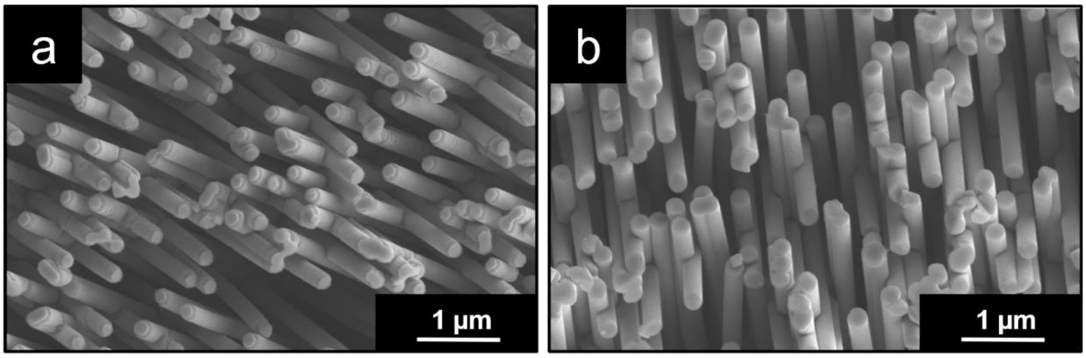

Figure S10. SEM images of the samples after electrochemical oxidation of Cu_2O nanowires using a solution with (a) PVP (5 $mg \cdot mL^{-1}$) and (b) benzoic acid (58 mM) but without the presence of BTC. The nanowire surface appears smooth (without nanoparticles), confirming that no MOF particles could grow as BTC was not used during the synthesis.

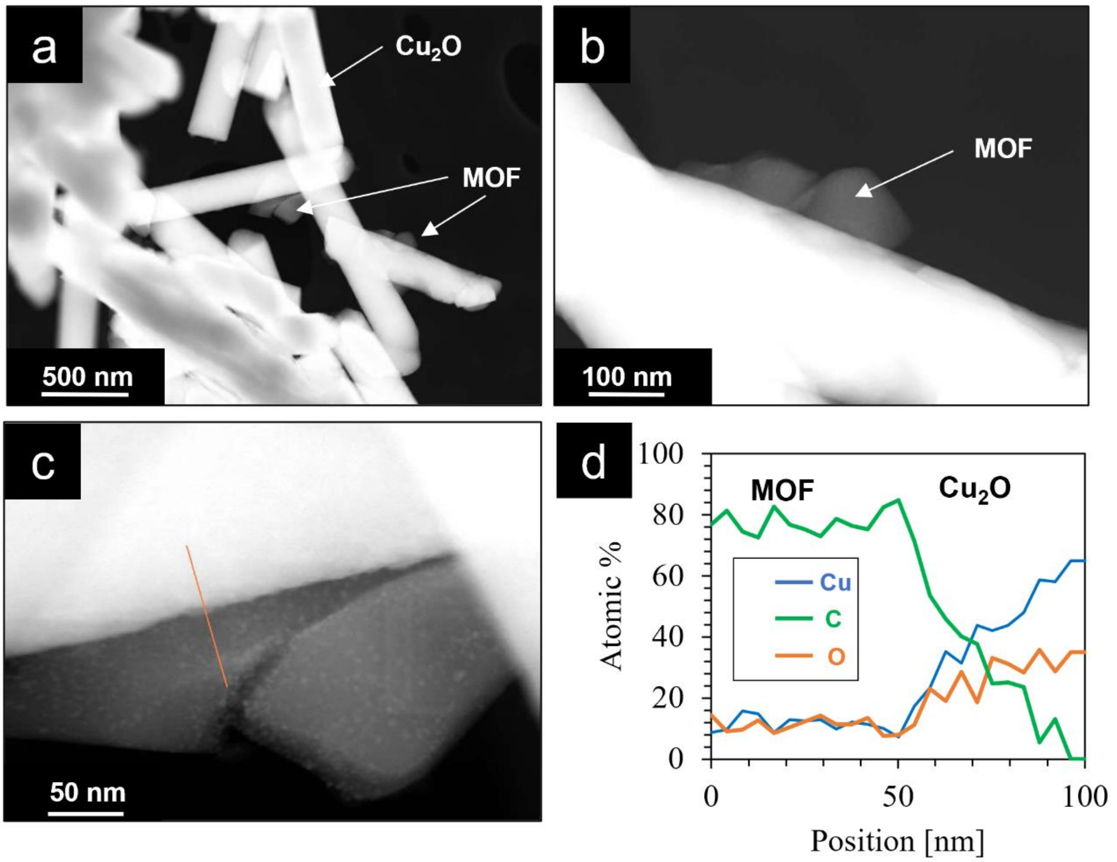

Figure S11. (a-c) LAADF-STEM images of Cu_2O nanowires after partial conversion into HKUST-1 using benzoic acid at different magnifications; (d) EDX line-scan across the MOF-Cu_2O interface as indicated in (c) showing an estimation of the amount of C, Cu and O (in atomic %) by Cliff-Lorimer quantification.

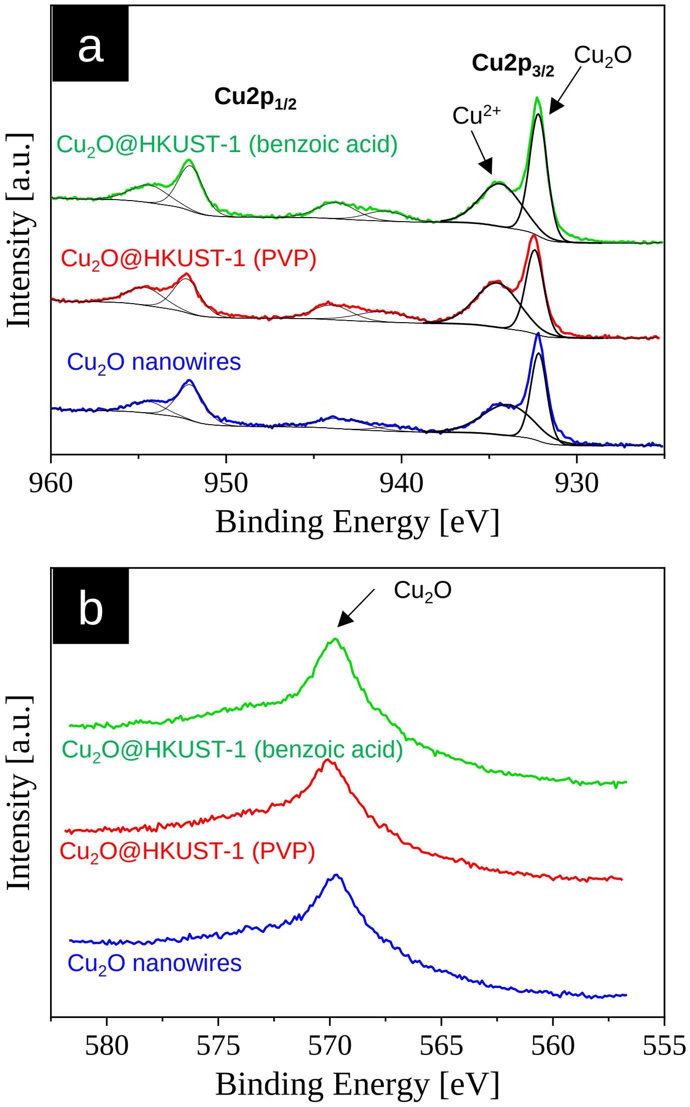

Figure S12. (a) Cu 2p and (b) Cu LM2 XPS spectra for the Cu_2O@HKUST-1 nanowires. The spectra show the presence of both Cu^+ and Cu^{2+} species for all the samples.
